# Pleiotropic expression quantitative trait loci are enriched in enhancers and transcription factor binding sites and impact more genes

**DOI:** 10.1016/j.csbj.2024.11.019

**Published:** 2024-11-17

**Authors:** Aitor González, Pascale Paul

**Affiliations:** Aix-Marseille Univ, INSERM U1090, TAGC, Marseille 13288, France

**Keywords:** Genome-wide associations studies (GWAS), expression quantitative trait loci (eQTL), Pleiotropy, Colocalization

## Abstract

Integrating expression quantitative trait loci (eQTL) data with genome-wide association studies (GWAS) enables the discovery of pleiotropic gene regulatory variants that influence a wide range of traits and disease susceptibilities. However, a comprehensive understanding of the distribution of pleiotropic QTLs across the genome and their phenotypic associations remain limited. In this study, we systematically annotated genetic variants associated with both trait variation and gene expression changes, focusing specifically on the unique characteristics of pleiotropic eQTLs. By integrating data from 127 eQTL studies and 417 traits from the IEU Open GWAS Project, we identified 476 pleiotropic eQTL variants affecting two or more distinct traits. Our analysis highlighted 5345 eQTL candidates potentially linked to gene expression changes across 293 GWAS traits. Notably, the 476 pleiotropic eQTLs associated with multiple trait categories were localized within a cumulative 2.5 Mbp genomic region. These pleiotropic eQTLs were enriched in enhancer regions and CTCF loops, influencing a larger number of genes in closer genomic proximity. Our findings reveal that pleiotropic eQTLs are concentrated within a small fraction of the genome and exhibit distinct molecular features. Colocalization results are accessible through an interactive web application and UCSC genome browser tracks at https://gwas2eqtl.tagc.univ-amu.fr/gwas2eqtl, facilitating the exploration of pleiotropic eQTLs and their roles in gene regulation and disease susceptibility.

## Graphical Abstract

**Figure:**
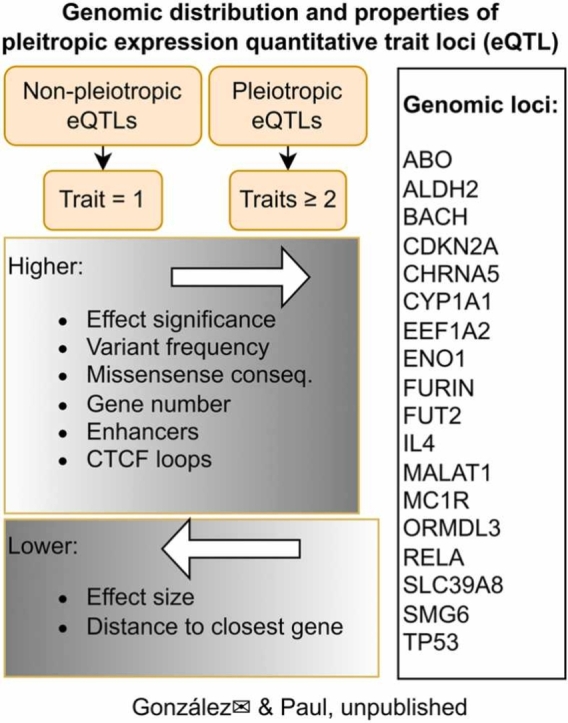

## 1 Introduction

The growing number of genome-wide association studies (GWAS) linking genetic variants to phenotypic or disease traits has led to the constant expansion of comprehensive databases such as the NHGRI-EBI GWAS Catalog [1]. As of 2019, the NHGRI-EBI GWAS Catalog included 5687 studies (GWAS), encompassing 71,673 variant-trait associations from 3567 publications. The ENCODE consortium allowed to define candidate cis-regulatory elements (CREs), which, combined with evolutionary conservation methods, help in identifying regulatory elements under evolutionary pressures [2]. Large scale GWAS studies have further revealed that many genetic variants exhibit pleiotropy, meaning a single gene can influence multiple, seemingly unrelated phenotypes or traits.

With the extensive availability of genomic data, it is now possible to investigate pleiotropy at various levels, including the variant level [3], [4], [5], the genomic region level [5], the gene level [6] and at the level of co-regulated gene groups [7].

Previous studies estimate that the human genome contains between 18,000 and 75,000 pleiotropic variants, 7757 pleiotropic genes, and cumulated pleiotropic regions ranging 180–1707 megabases (Mb) in the human genome [3], [4], [5]. These estimates arise from different GWAS approaches, which likely contain both false positives and false negatives [3].

Expression quantitative trait loci (eQTLs) are genetic variants associated with differences in the expression of one or more genes, thereby shaping gene activity in a cell- and tissue-specific manner [3]. Gaining knowledge on eQTLs can thus allow us to decipher the molecular mechanism that associates these variants to GWAS traits [8]. However, due to linkage disequilibrium, these methods may fail to identify the causal variant. Statistical colocalization of eQTLs and GWAS variants, as well as eQTLs annotation of GWAS traits has been explored [9]. While ColocDB includes extensive colocalization of eQTLs and GWAS variants, analysis to identify pleiotropic eQTLs is currently missing [10]. Because many functional common variants lie outside coding sequences, annotating GWAS variants with eQTLs is crucial to uncover the genes and tissues underlying GWAS traits, particularly for identifying the characteristics of pleiotropic eQTLs.

Previous research has shown that pleiotropic genomic regions and variants exhibit specific properties at the genomic and cellular levels, regulating a larger number of gene targets and showing activity in a broader range of tissues [5], [11], [12]. Pleiotropic variants also appear more frequent and exhibit higher effect sizes [3]. In this study, we leveraged two extensive eQTL and GWAS datasets from the EBI eQTL Catalogue [13] and the IEU OpenGWAS Project [14], respectively, to conduct a systematic colocalization analysis based on 127 eQTL studies and 417 GWAS across various cell types, tissues, and phenotypes. By categorizing these 417 traits, we identified pleiotropic variants as those associated with two or more categories. This categorization enabled us to develop an online application tool for exploring eQTL and GWAS colocalization, accessible at https://gwas2eqtl.tagc.univ-amu.fr/gwas2eqtl, allowing users to investigate colocalization patterns across diverse phenotypes and tissues.

## 2 Materials and methods

### 2.1 Summary statistics of expression quantitative trait Loci (eQTL) studies

We utilized data from 127 eQTL studies available in the EBI eQTL Catalogue, one of the most comprehensive resources providing uniformly processed eQTLs across diverse tissues and cell types[13] (Supplementary Table 1). We downloaded the summary statistics for these studies, representing 127 biological samples, from the EBI eQTL Catalogue To streamline analyses across heterogeneous cell types and tissues, we categorized these into 35 distinct groups (Supplementary Table 1). Tissue grouping was based on anatomical proximity, such as grouping various brain regions or segments of the digestive tract (e.g., colon). Circulating or immortalized cell types were categorized according to functional roles, resulting in classifications such as blood, immune system, and lymphoblastoid cells.

### 2.2 Summary statistics of genome-wide association studies (GWAS)

GWAS summary statistics were sourced from the IEU OpenGWAS Project, which aggregates data from several databases, including the EBI's comprehensive GWAS database, manually curated GWAS datasets, and the UK Biobank [14]. We selected GWAS traits from the IEU OpenGWAS Project based on the following criteria: (1) exclusion of molecular traits (e.g., proteomic or methylome data) to focus on colocalization of eQTLs with disease-related variants; (2) inclusion of studies with European ancestry samples, aligning with the population represented in the EBI eQTL Catalogue; (3) stringent definitions of medical or physiological conditions, with environmental or self-reported traits excluded due to limited tissue linkage; and (4) studies with sample sizes of at least 10,000 participants, including a minimum of 2000 cases and 2000 controls. After applying these filters, 417 GWAS traits remained (see Supplementary Table 2), covering 10,621 clumped lead variants with p-values below 5 × 10⁻⁸ from 335 GWAS, which were used to annotate eQTLs.

### 2.3 Additional genomic datasets, tools

This study incorporated additional datasets and tools. Besides eQTL and GWAS datasets, we used transcription factor ChIP-seq peaks and cis-regulatory modules from the ReMap database [15], UCSC annotation data [16], and candidate enhancers ENCODE project’s SCREEN database (https://screen.encodeproject.org/) [17]. CTCF ChIA-PET loop data were also downloaded from ENCODE as bedpe files. Data exploration was conducted using the UCSC Genome Browser and the OMIM database (https://omim.org). and Pipelines were executed using Snakemake, which streamlined and automated the workflows [18].

### 2.4 Colocalization analysis

For the colocalization analysis, complete association data were obtained from OpenGWAS and converted to hg38 coordinates using Picard and Crossmap [19]. Top hit variants from the corresponding GWAS were selected based on a p-value threshold of 5 × 10⁻⁸ , using a clumping parameter of r² = 0.1 within a 1 Mb clumping window. Within a 1 Mb radius of these top hits, significant eQTL and GWAS variants with a p-value under 5 × 10⁻⁸ were extracted. Missing allele frequencies were filled in using data from the European population in the 1000 Genome Project [20].

Variants meeting various criteria, variant frequency strictly between 0 and 1, non-duplicated, and without missing data, were retained. Colocalization between eQTL and GWAS variants was assessed using the "coloc.abf" function from CRAN coloc package. Colocalization between eQTL and GWAS variants was assessed using the coloc.abf function from the CRAN coloc. This analysis was conducted for each window, eQTL sample, and GWAS. The coloc software was specifically utilized to calculate the probabilities that a variant is associated with two distinct traits simultaneously[9]. We prioritized the coloc software to leverage the R interface between our datasets and the tools provided by the IEU OpenGWAS Project. Throughout this paper, analyses were performed with a cutoff of PP.H4.abf≥ 0.75 and SNP.PP.H4 ≥ 0.5, unless explicitly stated otherwise. The parameter PP.H4.abf represents the posterior probability that there is a shared causal variant affecting both traits (e.g., GWAS and eQTL) at a given locus. The parameter SNP.PP.H4 is the posterior probability for an individual variant at that locus to be the shared causal variant. We adopted PP.H4.abf≥ 0.75 to facilitate comparison with a previous large colocalization analysis using the same software [8].

### 2.5 Statistical analysis

For statistical analyses, we used the Fisher's exact test, the Mann-Whitney U test, the odds ratio test and the Spearman correlation. We set the following significance levels: ns (not significant) ≥ 0.05; * < 0.05; ** < 0.01; *** < 0.001; *** * < 0.0001. Details of the statistical tests are provided in the figure legends.

## 3 Results

### 3.1 Colocalization of variants associated with GWAS traits and gene expression changes

We developed a comprehensive pipeline for colocalization of variants from GWAS and eQTL studies (see Section Methods for details). Variants within the MHC locus (chromosome 6: 25,000,000–35,000,000 bp, hg38) were excluded from this analysis due to their complex linkage disequilibrium structure. The pipeline resulted in colocalization between traits and expression associations for 138,274 variants derived from 293 GWAS and 127 eQTL studies, applying a cutoff value of PP.H4.abf ≥ 0.75 (see Data availability for details). Following the selection of individual variants at the locus with moderate probability (SNP.H4. PP≥ 0.5) of being the shared causal variant, we retained 5345 variants and 7040 eQTL genes (Supplementary Table 4). The cutoff values of PP.H4.abf≥ 0.75 and SNP.H4. PP≥ 0.5 were selected based on a previous colocalization analysis conducted by Mu et al. [9]. These 5345 variants were used for subsequent analyses.

### 3.2 Differential contribution of eQTLs to GWAS variant interpretation across disease categories

**Fig. 1:**
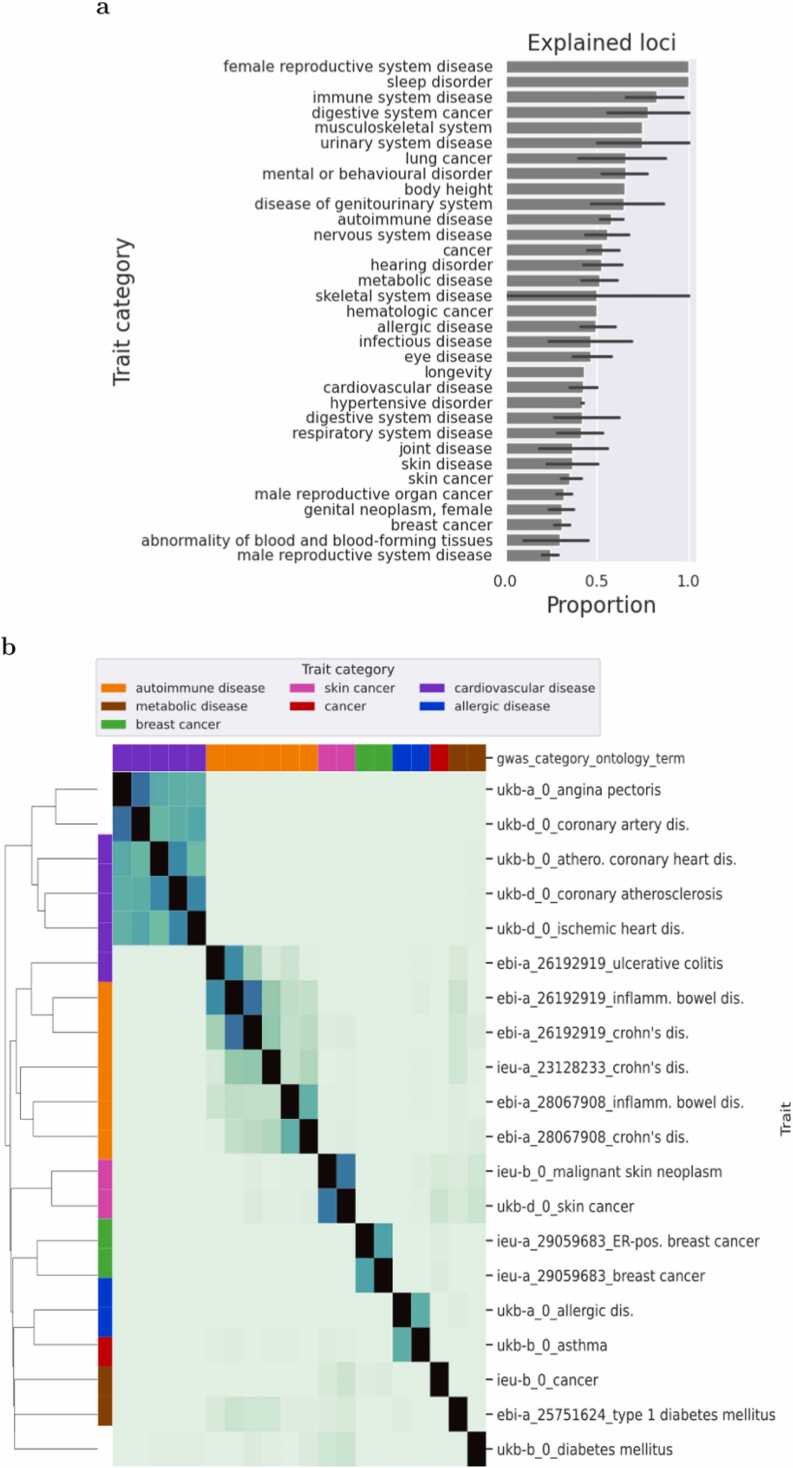
Proportion of explained GWAS leading loci and trait clustering based on eQTLs. a) The average proportion of explained loci in GWAS across different trait categories is illustrated. Detailed proportions can be found in Supplementary Table 3, with error bars representing the 95 % confidence level. b) The distance between traits is determined based on the Spearman correlation between eQTL coefficients. Only GWAS traits with a minimal number of eQTLs between them were selected. Labels include the dataset source (ebi-a: Datasets meeting minimum requirements imported from the EBI database of complete GWAS summary data; ieu-a: GWAS summary datasets generated by various consortia, initially developed for MR-Base; ukb-a: Neale lab analysis of UK Biobank phenotypes, round 1; ukb-b: IEU analysis of UK Biobank phenotypes; ukb-d: Neale lab analysis of UK Biobank phenotypes, round 2), the PubMed identifier (if available, otherwise 0), and the trait ontology term.

In our analysis, we aimed to characterize disease variants by leveraging the extensive number of co-localized eQTLs and GWAS variants identified earlier. Specifically, we assessed the proportion of leading GWAS variants that could be elucidated by colocalization with eQTLs within a 1 Mbp window (Supplementary Table 3). Our findings demonstrated significant variability, with between 25 % and 80 % of leading GWAS variants being explained by eQTLs. Notably, the top two categories—sleep disorders and female reproductive system diseases—showed complete explanation by eQTLs; however, these values were derived from only one GWAS with few loci (Fig. 1a, Supplementary Table 3). Overall, the extent to which eQTLs explain GWAS variants varies markedly, ranging from 25 % to 100 % across different disease categories.

### 3.3 Web application for exploring pleiotropic variations associated with traits and gene expression

To improve accessibility to this valuable resource, we developed a web application for exploring colocalized eQTLs and GWAS traits (https://gwas2eqtl.tagc.univ-amu.fr/gwas2eqtl/). This tool allows users to investigate pleiotropic variants associated with a diverse array of traits, including autoimmune diseases, breast cancer, cardiovascular diseases, and height. Additionally, we provide a UCSC track featuring annotated variants (see Data Availability). Supplementary Fig. 1a-c presents three screenshots of these UCSC tracks for B-cells, monocytes, and frontal cortex biological samples, all mapped to chromosome 5 around the IL4 gene.

Importantly, the UCSC track illustrates that associated eQTLs and genes vary significantly based on cell type and tissue distribution. For example, in B-cells, identified eQTLs predominantly target the SLC22A5 and MIR3936HG genes (see Supplementary Fig. 1a). In monocytes, eQTLs also target P4HA2 and SLC22A4 genes (see Supplementary Fig. 1b). In contrast, eQTLs in the prefrontal cortex predominantly affect PDLIM4 and RAD50 (see Supplementary Fig. 1c). This variation underscores that the gene targets of eQTLs co-localizing with different GWAS traits exhibit significant differences depending on the specific cell type.

### 3.4 Coherent clustering of traits based on eQTL effects

In the preceding sections, we annotated variants based on associated traits and gene expression. Here, we aim to check if different traits cluster coherently based on the eQTL effects of co-localized eQTL/GWAS variants. To achieve this, we manually categorized 417 GWAS traits into 35 groups, clustering identical or similar traits (see Supplementary Table 2). For each trait, we extracted the beta effect values of various co-localized eQTLs (PP.H4.abf ≥ 0.75 and SNP.PP.H4 ≥ 0.5) across different tissues and computed distances between the vectors of different traits using Spearman correlation.

To refine the number of traits and categories, we retained only those traits that showed a correlation of at least 0.05 or −0.05 with at least 30 other traits. This analysis revealed coherent clustering among traits related to autoimmune diseases, circulatory system disorders, cancers, and allergies, indicating that similar diseases share common eQTLs (see Fig. 1b). Notably, skin cancer exhibited overlapping signals with autoimmune diseases, such as type 1 diabetes and digestive autoimmune disorders (see Fig. 1b), suggesting potential comorbidities.

### 3.5 Identification of eQTLs associated with two or more trait categories

In the previous section, we categorized traits into distinct groups and postulated that these categories could be utilized to identify pleiotropic eQTLs associated with multiple trait categories. We annotated eQTLs according to these trait categories, categorizing them based on the number of categories they encompass. Using a cutoff of SNP.PP.H4 ≥ 0.5, we identified 476 pleiotropic eQTLs linked to two or more categories (refer to Supplementary Table 4). Notably, the most pleiotropic eQTLs were associated with categories covering a range of traits, including autoimmune diseases, circulatory diseases, and cancer (see Table 1). These eQTLs are located near genes such as ALDH2 (12q24.12), ENO1 (1p36.23), ORMDL3 (17.q21.1), IL4 (5q31.1), BACH2 (6q15), FUT2 (19q13.33), SLC39A8 (4q24), and CYP1A1 (15q24.1) (see Table 1). Supplementary Figure 1 illustrates the genomic region around the IL4 gene, highlighting highly pleiotropic eQTLs such as rs2522051 and rs17622656 along with their target genes.

**Table 1:** Pleiotropic eQTLs in various cytobands associated with four distinct trait categories.

Chrom.	Pos. (hg38)	Cytoband	rsid	eQTL gene marker	Trait categories								
1	8,437,247	1p36.23	rs301802	ENO1	allergic disease; cardiovascular disease; hypertensive disorder; mental or behavioural disorder								
4	102,267,552	4q24	rs13107325	SLC39A8	autoimmune disease; cardiovascular disease; hypertensive disorder; musculoskeletal system								
5	132,461,886	5q31.1	rs2522051	IL4	breast cancer; cardiovascular disease; hypertensive disorder; respiratory system disease								
6	90,267,049	6q15	rs72928038	BACH2	allergic disease; autoimmune disease; metabolic disease; skin cancer								
12	111,621,753	12q24.12	rs11065979	ALDH2	allergic disease; cardiovascular disease; eye disease; longevity								
15	74,770,056	15q24.1	rs11072508	CYP1A1	cardiovascular disease; hypertensive disorder; immune system disease; joint disease								
17	39,895,095	17q21.1	rs8067378	ORMDL3	allergic disease; autoimmune disease; cardiovascular disease; metabolic disease								
19	48,703,417	19q13.33	rs601338	FUT2	autoimmune disease; cardiovascular disease; digestive system disease; hypertensive disorder								

The term "eQTL gene marker" denotes the most frequently cited gene within the eQTL target genes.

In a comparative analysis with another study on pleiotropy, where trait categories were aggregated based on genomic proximity, we found that the eQTLs identified in our study were associated with significantly more categories (see Supplementary Fig. 2a) [5]. Further examination demonstrated that for eQTLs associated with one, two, and more categories, 10–15 % of our eQTLs overlapped with those from the previous study (refer to Supplementary Fig. 2b). In conclusion, we identified 476 pleiotropic eQTLs associated with a diverse array of diseases, including autoimmune diseases, cardiovascular disorders, hypertensive disorders, mental or behavioral conditions, and musculoskeletal disorders.

### 3.6 Specificity of eQTLs across traits, gene targets, and tissues

In our analysis, we classified eQTLs based on the number of trait categories and explored the relationship between this count and the number of target genes and tissues. We aimed to determine whether eQTLs exhibit specificity to particular traits, gene targets, and tissues. We computed the proportion of eQTLs annotated with various counts of trait categories, gene targets, and tissues.

**Fig. 2:**
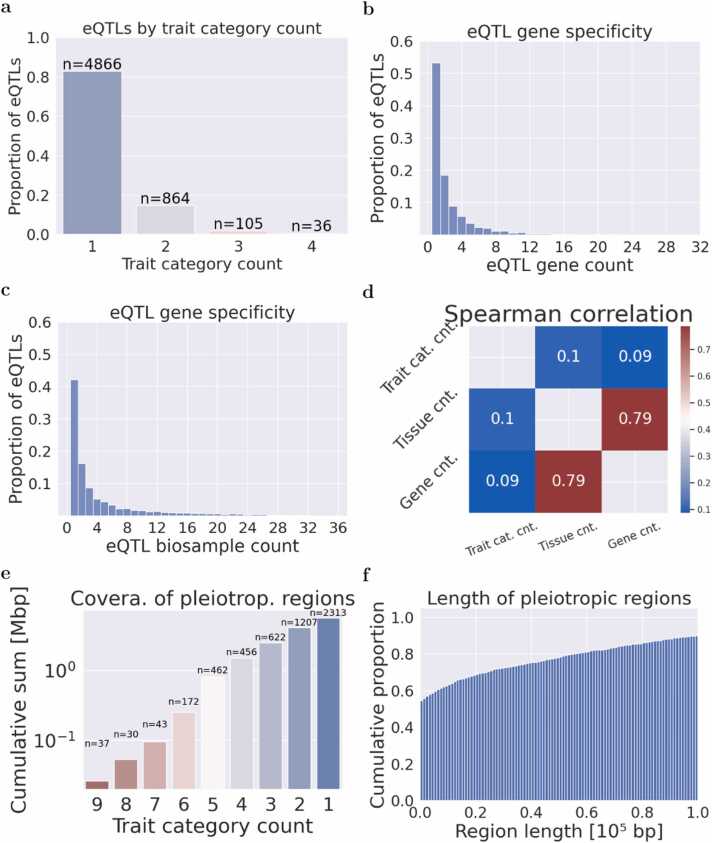
Distribution of eQTLs and regions based on trait counts, eQTL gene specificity and biological samples. a-c) The proportion of eQTLs is examined based on the count of trait categories (a), eQTL genes (b), and eQTL biological samples (c). The count of eQTLs is shown over the bars in Figure (a). d) The Spearman correlation between the count of trait categories, eQTL genes, and eQTL biological samples is explored for colocalized eQTLs and GWAS variants. “cnt” stands for count. e, f) To merge colocalized variants to pleiotropic regions, we iteratively merge colocalized variants located at less than 100,000 bp into regions and annotate the regions with the number of trait categories. e) This plot shows the cumulative length of the regions containing colocalized variants starting with the most pleiotropic categories. The number above the bar shows the number of pleiotropic eQTLs. f) This plot shows the cumulative proportion of genomic regions containing eQTLs associated to two or more trait categories with different lengths. Around 60 % of these regions show a length of 10,000 bp or less.

The results indicate that the majority of eQTLs (approximately 80 %) are associated with a single trait category, while about 10 % are linked to two trait categories, and less than 1 % to three or more (see Fig. 2a). Repeating this analysis for eQTLs of each trait category revealed that around 80 % of eQTLs related to allergic diseases are involved in one category, compared to only 60 % for those in cardiovascular diseases, which are associated with two or more categories (see Supplementary Fig. 3a and 3b). This suggests that eQTLs in cardiovascular-related diseases tend to be more pleiotropic. Regarding gene targets, approximately 50 % of eQTLs modify the expression of a single gene, 20 % affect two genes, and the remaining eQTLs influence three or more genes (see Fig. 2b). Concerning tissues, around 40 % of eQTLs are found in a single tissue, approximately 20 % in two tissues, and the rest in three or more (see Fig. 2c).

We further explored the correlation between the number of GWAS categories, genes, and tissues. The counts of eQTL genes and tissues showed a high correlation (r = 0.79), while the count of trait categories exhibited a weaker correlation with both gene and tissue counts (see Fig. 2d). In summary, most eQTLs are associated with a single trait category, although these eQTLs regulate multiple genes across various tissues.

### 3.7 Concentration of pleiotropic eQTLs in 2.5 Mbp cumulated genomic regions

Our investigation into pleiotropic eQTLs revealed that these loci are concentrated in specific regions of the genome. Notably, cytobands such as 3q23, 5q31.1, 9p21.3, 15q24.1, and 19q13.33 emerge as key locations for the most pleiotropic eQTLs (see Table 1 and Supplementary Table 4). This observation led us to explore whether a small fraction of the genome contains the majority of pleiotropic eQTLs.

To identify genomic regions associated with pleiotropic eQTLs, we used a sliding window of 100,000 bp, aggregating regions containing pairs of co-localized eQTL variants annotated with two or more categories, located within a maximal distance of 100,000 bp. Each eQTL was assigned to a single region. The complete list of regions with annotations is provided in Table 2 and Supplementary Table 5. Our findings indicate that regions with five or more trait categories encompass approximately 0.8 Mbp of the genome, while regions with four trait categories total 1.5 Mbp, and those with three or more trait categories account for 2.5 Mbp (see Fig. 2e). Additionally, regions containing at least two trait categories and measuring less than 10,000 bp constitute around 60 % of the total pleiotropic regions, whereas regions up to 100,000 bp comprise 90 % of the total pleiotropic regions (see Fig. 2f).

**Table 2:** Regions exhibiting pleiotropy involving six or more trait categories.

Chrom.	Start (hg38)	End (hg38)	Cytoband	eQTL gene marker	Trait categories	Length	eQTL count
5	132,239,645	132,497,907	5q31.1	IL4	allergic disease; autoimmune disease; body height; breast cancer; cancer; cardiovascular disease; hypertensive disorder; respiratory system disease; skin disease	258,263	37
9	21,950,524	22,207,038	9p21.3	CDKN2A	breast cancer; cancer; cardiovascular disease; eye disease; genital neoplasm, female; male reproductive organ cancer; skin cancer	256,515	9
9	133,242,881	133,278,537	9q34.2	ABO	abnormality of blood and blood-forming tissues; cardiovascular disease; eye disease; genital neoplasm, female; infectious disease; mental or behavioural disorder	35,657	9
11	65,488,118	65,638,129	11q13.1	NEAT1	cardiovascular disease; eye disease; hypertensive disorder; joint disease; metabolic disease; respiratory system disease	150,012	8
11	65,747,403	65,909,045	11q13.1	RELA	allergic disease; autoimmune disease; breast cancer; cardiovascular disease; hypertensive disorder; skin cancer; skin disease	161,643	34
15	74,751,897	74,843,920	15q24.1	CYP1A1	breast cancer; cardiovascular disease; hearing disorder; hypertensive disorder; immune system disease; joint disease	92,024	15
15	78,428,581	78,762,558	15q25.1	CHRNA5	breast cancer; cardiovascular disease; longevity; lung cancer; mental or behavioural disorder; respiratory system disease	333,978	29
15	90,861,475	91,070,064	15q26.1	FURIN	breast cancer; cardiovascular disease; genital neoplasm, female; hypertensive disorder; mental or behavioural disorder; metabolic disease	208,590	38
16	89,626,691	89,808,935	16q24.3	MC1R	breast cancer; cancer; cardiovascular disease; eye disease; hypertensive disorder; skin cancer	182,245	24
17	2006,529	2311,037	17p13.3	SMG6	allergic disease; cardiovascular disease; eye disease; hypertensive disorder; mental or behavioural disorder; respiratory system disease	304,509	22
17	7452,302	7718,459	17p13.1	TP53	cancer; cardiovascular disease; hypertensive disorder; joint disease; respiratory system disease; skin cancer	266,158	27
20	63,588,387	63,857,282	20q13.33	EEF1A2	allergic disease; autoimmune disease; cancer; cardiovascular disease; hypertensive disorder; male reproductive organ cancer; metabolic disease; skin disease	268,896	30

These regions were constructed around pleiotropic eQTLs linked to two or more trait categories using a sliding window of 100,000 base pairs. The "eQTL gene marker" column indicates the most cited eQTL gene in the region, as per NCBI PubMed. The "eQTLs cnt." column provides the count of eQTLs in that region. Genomic coordinates are presented for the hg38 assembly.

The most pleiotropic region, associated with nine trait categories, is located at 5:131,912,097–132,802,472 in cytoband 5q31.1, encompassing genes such as the interferon response factor 1 (IRF1) and interleukins IL3, IL4, IL5, and IL13 (refer to Table 2). The largest region, spanning 7:2,712,518–7,254,268 in cytoband 7p22.3, measures 4,541,751 bp and contains variants linked to autoimmune diseases, respiratory diseases, and height (see Supplementary Table 5). This analysis underscores that pleiotropic eQTLs are highly concentrated in a small fraction of the genome.

### 3.8 Pleiotropic eQTLs exhibit lower effects on traits, increased significance, and higher variant frequencies

Building on previous studies indicating that pleiotropic variants tend to have lower effects and higher frequencies [3], our analysis explored how the effects (beta), association significance, and variant frequencies vary based on the number of trait categories associated with pleiotropic eQTLs.

**Fig. 3:**
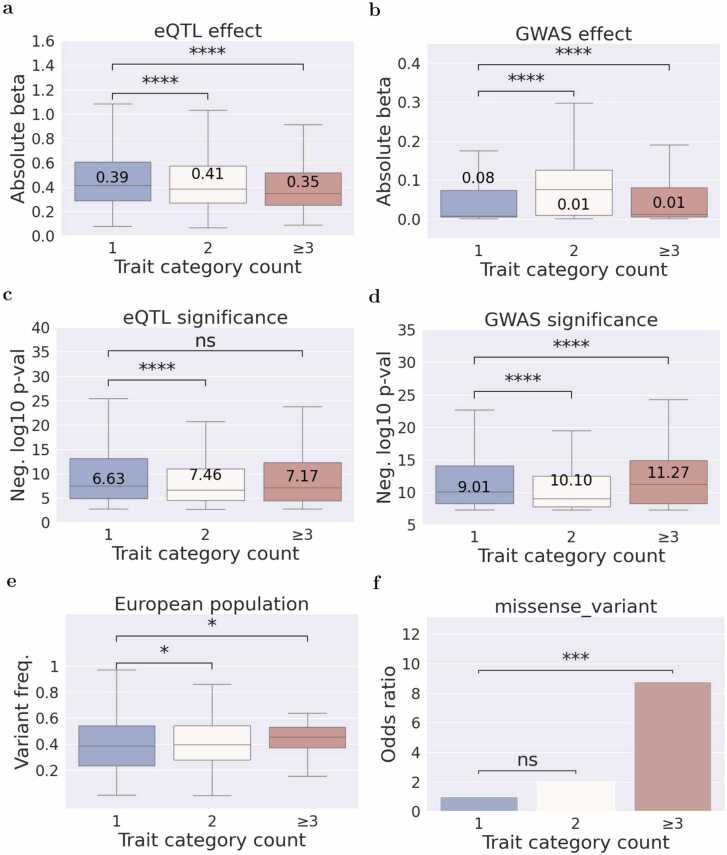
Properties of eQTLs across various pleiotropy levels: insights into variant effects on gene expression, trait associations, and allele frequencies. a, b) Average of the absolute effect of variants on gene expression (a) and traits (b). c, d) Average of the negative log10 of the p-value of the association with gene expression (c) and traits (d). e) Average variant frequency in the European population from the 1000 Genomes database. This plot gives the distribution of variant frequencies of colocalized eQTL/GWAS variants partitioned by the trait category count. g) Odds ratio of the eQTLs annotated with the given ENSEMBL variant effect predictor compared to eQTLs with a single trait category count. Median values are shown in the boxes of figures a-d. Boxes of the boxplots show the quartiles of the dataset while the whiskers extend to show the rest of the distribution except outliers. In figures a-e, we carried out the Mann-Whitney U test with a two-sided research hypothesis, and in figure f, the Fisher exact test with a two-sided research hypothesis (Significance: ns ≥0.05 * <0.05, ** <0.01, *** <0.001, **** <0.0001).

We analyzed the average absolute eQTL and GWAS effect sizes (beta) in relation to the increasing number of trait categories. We observed that the effect size on gene expression is significantly different, although the direction of the changes is inconsistent across varying levels of pleiotropy (see Fig. 3a). In contrast, the effect size on the GWAS trait clearly decreases with increasing eQTL pleiotropy (see Fig. 3b). Further examination of the significance of eQTL and GWAS effects revealed a notable increase for both gene expression and GWAS traits (see Fig. 3c, d).

Comparing average effect sizes from different studies can be challenging, as these depend on study size and variant frequencies. To homogenize these variables, we repeated the comparison of eQTL and GWAS effects using variants with restricted frequencies (0.45–0.55) or sample sizes (75,000−125,000) (see Supplementary Fig. 4). These results confirmed the previous observations that GWAS effects decrease in pleiotropic eQTLs.

We also explored the relationship between variant frequency and pleiotropy. We partition variant frequencies of each population by the trait category count. The variant frequencies of pleiotropic eQTLs were significantly higher in the European population (see Fig. 3e). In other populations, we did not observe a significantly higher variant frequency, potentially influenced by the fact that colocalization was computed in the European population (Data not shown). In summary, our analysis suggests that pleiotropic eQTLs exhibit lower effects on traits, are more significant, and are more frequent in the European population.

### 3.9 Pleiotropic eQTLs are more likely missense variants

To gain insights into the variant effect consequences of pleiotropic eQTLs compared to non-pleiotropic eQTLs, we utilized the EBI variant effect predictor (VEP) for annotation [21]. Subsequently, we calculated odds ratios and performed Fisher exact tests for pleiotropic eQTLs. The analysis revealed that pleiotropic eQTLs exhibit significantly higher odds ratios for being missense variants, with values of two and nine for category counts of two and more, respectively. This finding strongly suggests that pleiotropic eQTLs are more likely to be associated with missense variants.

### 3.10 Pleiotropic eQTLs are closer to gene targets

**Fig. 4:**
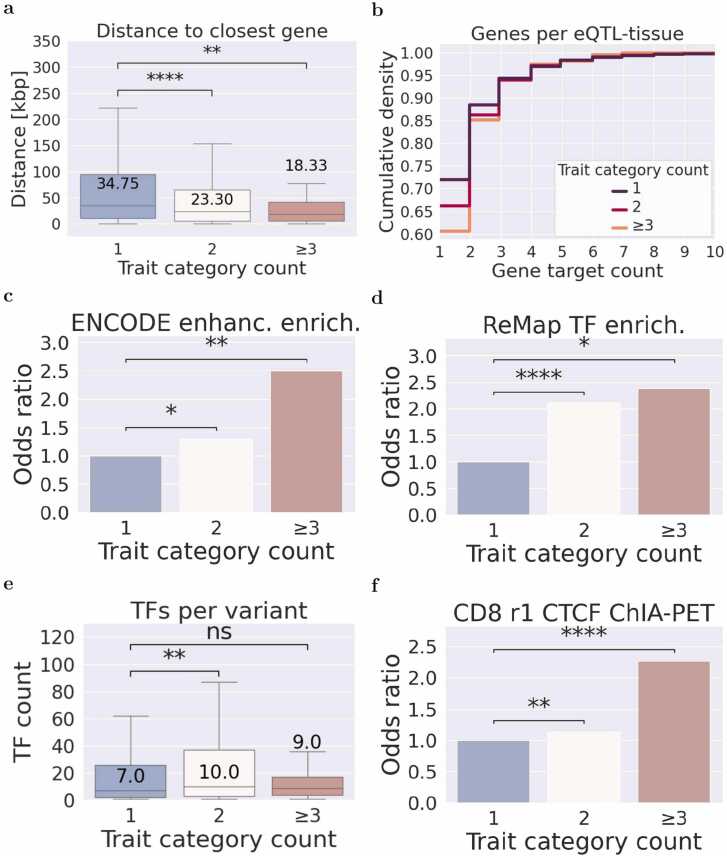
Exploring eQTL gene distance and biological sample counts, transcription tactor binding, and CRM annotation across diverse pleiotropy levels. a) Closest gene distance for each eQTL in relation to the count of trait categories. b) Cumulative proportion of eQTL-tissue pairs for increasing counts of eQTL target genes partitioned by trait category counts. c) Odds ratio of eQTLs annotated with ENCODE enhancer versus non-annotated regions for different trait category counts. d) Odds ratio of eQTLs annotated with ReMap non-redundant transcription factor peaks versus non-annotated regions for different trait category counts. e) Count of bound transcription factors (TF) in the region surrounding eQTLs with a radius of 10 bp (Window 20 bp) for different trait category counts. f) Odds ratio of eQTLs within CTCF ChIA-PET loops in isogenic replicate r1 of CD8 T-cells for different trait category counts. Boxes of the boxplots depict the quartiles of the dataset, while the whiskers extend to show the rest of the distribution except outliers. In Figs. a and c, we conducted the Mann-Whitney U test, with a two-sided research hypothesis, and in figure d, the Fisher exact test with a one-sided research hypothesis (Significance: ns ≥0.05 * <0.05, ** <0.01, *** <0.001, **** <0.0001).

In our exploration of pleiotropic eQTLs, we delved into understanding their spatial relationship with gene targets, focusing on the distance to the closest and the most distal gene. The analysis revealed that the median distance of eQTLs associated with one, two, and more than two trait categories to their closest gene is 35 kbp, 23 kbp, and 18 kbp, respectively (see Fig. 4a). This suggests a significant reduction in the distance of pleiotropic eQTLs to their closest gene targets. However, when analyzing the distance of pleiotropic eQTLs to the furthest gene target, no significant difference was found (Data not shown). This indicates that the distance of the eQTL to its furthest gene target remains relatively consistent in pleiotropic eQTLs. In summary, our analysis suggests that pleiotropic eQTLs exhibit a shorter distance to their closest gene targets, potentially reflecting a more direct influence on gene regulation.

### 3.11 Pleiotropic eQTLs have lower gene specificity

In our investigation into the characteristics of pleiotropic eQTLs, we turned our attention to their relationship with the number of target genes. Our analysis involved computing the cumulative proportion of eQTLs associated with different numbers of target genes in specific tissues. The findings revealed that the proportion of eQTLs targeting only one gene was approximately 0.72, 0.65, and 0.6 for eQTLs associated with one, two, and more than two trait categories, respectively (see Fig. 4b). This trend was similarly observed for eQTLs targeting two genes. This observation could be related to the higher variant frequency of pleiotropic eQTLs. To homogenize variant frequencies, we repeated this analysis with variants that show a frequency in the European population between 0.45 and 0.55 (Supplementary Fig. 5b). Both analyses suggest that pleiotropic eQTLs tend to modulate a larger number of genes, indicating a broader impact on gene regulation compared to non-pleiotropic eQTLs.

### 3.12 Cumulative proportion analysis reveals lower tissue specificity of pleiotropic eQTLs

Continuing our exploration of pleiotropic eQTLs, we turned our focus to their relationship with the number of tissue categories. Our analysis involved computing the cumulative proportion of eQTLs targeting genes in each number of tissues.

The observations revealed systematic higher proportions of non-pleiotropic eQTLs in a low number of tissues (Supplementary Fig. 5a). This observation could be related to the higher variant frequency of pleiotropic eQTLs. To homogenize variant frequencies, we repeated this analysis with variants that show a frequency in the European population between 0.45 and 0.55 (Supplementary Fig. 5c). Both analyses suggest that pleiotropic eQTLs exhibit a tendency to be less tissue-specific, influencing gene expression across a broader range of tissues compared to their non-pleiotropic counterparts.

### 3.13 Enhancer properties of pleiotropic eQTLs

In our quest to understand the characteristics of pleiotropic eQTLs, we explored their association with enhancer regions and the binding of transcription factors (TFs). Utilizing the ReMap database [15], we examined the number of unique transcription factors bound within a radius of 10 bp around each eQTL, using a window of 20 bp. We show that pleiotropic eQTLs associated to two and three or more trait categories are significantly enriched in ENCODE enhancer regions with odds ratios 1.5 and 2.5, respectively (Fig. 4c). We also find that pleiotropic eQTLs associated to two and three or more trait categories are significantly enriched in peaks of ReMap transcription factors with odds ratios 1.5 and 2.5, respectively (Fig. 4d). These two results suggest that gene regulatory regions show more robust properties for pleiotropic eQTLs. We show that eQTLs associated with one, two, or more trait categories exhibit a median binding of 7, 10, and 9 transcription factors, respectively (Fig. 4e). This suggests that pleiotropic eQTLs tend to be associated with a higher number of TFs.

Enhancers are likely enclosed within CTCF loops [22]. To investigate the relationship of pleiotropic eQTLs/gene links and CTCF loops, we evaluated the enrichment of the regions linking pleiotropic eQTLs and target genes withing CTCF ChIA-PET loops in several cell lines from ENCODE. Our results shows that the links between pleiotropic eQTLs and target genes are enriched in the CTCF loops of cell lines A549 (Adenocarcinomic alveolar basal epithelial cells), CD8-T-cells, GM10248 (Lymphoblastoid cell line), HUVEC (Human umbilical vein endothelial cells), K562 (Erythroleukemia) and WTC11 (Human induced pluripotent stem cell) (Fig. 4f and Supplementary Fig. 6).

We investigated the presence of cis-regulatory modules (CRMs), which are non-coding genomic regions with a higher density of bound transcription factors [15]. The odds ratio of variants annotated with CRMs versus non-annotated was 1, 1.5, and 1.9 for eQTLs associated with one, two, or more trait categories, respectively (see Fig. 4d). This implies that pleiotropic eQTLs are not only bound by more transcription factors but are also more likely to be in cis-regulatory modules.

## 4 Discussion

Genome-wide association studies have uncovered numerous loci linked to various diseases, however the specific causal contributions of pleiotropic variants within these loci remain largely elusive. This study leveraged on two extensive databases, the IEU OpenGWAS project and the EBI eQTL Catalogue, encompassing summary statistics from 417 GWAS and 127 eQTL datasets, to conduct a comprehensive colocalization pipeline. Our objective was to clarify the characteristics of pleiotropic eQTLs, contributing to a more precise understanding of these variants.

These findings are accessible via an interactive web platform https://gwas2eqtl.tagc.univ-amu.fr/gwas2eqtl/, enabling users to explore these colocalizations and analyze the dataset, thus providing a valuable resource. Recently, COLOCdb, a database of colocalization data across 3000 GWAS and 13 xQTL types, has been published but this database was not used to systematically investigate the properties of human genetic variants [10].

Our study revealed that approximately 50 % of the prominent variants associated with autoimmune diseases can be elucidated by eQTLs. This percentage surpasses previous studies reporting 25–38 % explanatory power using eQTLs and splicing QTLs (sQTLs) [8], [23]. This explanatory capacity may stem from our broader inclusion of 127 eQTL datasets. In contrast, prior studies, such as Mu et al. [8], used a more limited immune cell types fore eQTLs and sQTLs potentially missing contributions in other tissues. Similarly, Connally et al. focused on breast tissue-sepecific eQTLs to study breast cancer, possibly overlooking immune cell interactions involved in cancer biology [24]. Our study suggests that colocalization studies may lack the breadth of eQTL coverage necessary to to fully account for GWAS findings across diverse traits [25].

Our colocalization results also demonstrate remarkable consistency across independent datasets. For instance, related autoimmune diseases affecting the digestive system, such as inflammatory bowel disease, Crohn’s disease, and ulcerative colitis, showed strong colocalization coherence, despite originating from distinct GWAS sources [25], [26], [27]. Our analysis extended to predict overlaps with other autoimmune diseases, including type 1 diabetes, and traits associated with digestive autoimmune and inflammatory diseases, and skin cancer. These findings underscore the potential of pleiotropic eQTLs in drug repurposing [28].

Additionally, our data highlights the stability of cell-specific eQTL patterns across different independent datasets. Although the effects of quantitative trait loci (QTLs) vary widely between cell types, specific cell types maintain consistent patterns across studies. For example, the colocalization results for eQTLs targeting the gene SLC22A5 in B-cells exhibited similar results across three different studies [29], [30], [31]. This consistency is was also observed in monocytes and cortex samples.

Our analysis of pleiotropic eQTLs across varying SNP.PP.H4 cutoff values (0.25, 0.5, and 0.75) confirmed stable identification of key variants. High-pleiotropy variants, such as rs11065979, rs11072508, rs13107325, rs301802, rs2522051, rs601338, and rs72928038, were consistently detected at all three cutoff values. An intermediate cutoff value of 0.5 was chosen to strike a balance between sensitivity and specificity in identifying colocalized eQTLs.

The functional relevance of identified pleiotropic variants aligns with current literature. For instance, rs13107325, a pleiotropic eQTL associated with the SLC39A8 gene, has been validated in knock-in mouse models [32]. Similarly, the physiological impact of rs72928038 on the BACH2 gene was recently confirmed in b-cell lines and mutant mice [33].

Among the most pleiotropic regions, such as those around ABO, CDKN2A, CHRNA5, CYP1A1, EEF1A2, FURIN, IL4, MC1R, and TP53, the selection of regions with pleiotropic eQTLs remained insensitive to changes in the SNP.PP.H4 cutoff value. Notably, genes like ABO, CDKN2A, and FURIN, known for their pleiotropic effects on a wide array of phenotypes, were consistently identified [33], [34], [35].

In alignment with prior reports, we observed that pleiotropic eQTLs tend to be less tissue-specific [3], [5]. Additionally, our findings indicate that pleiotropic eQTLs are less gene-specific, aligning with previous studies demonstrating that eQTLs affecting multiple neighboring genes exhibit greater pleiotropy [12]. Mechanistically, this aligns with the notion that eQTLs influencing a higher number of genes and active in more tissues are associated with a broader range of traits. Pleiotropic variants were also found to be more frequent, a trend that might be a statistical artifact, where more frequent variants are seemingly more likely to be associated with specific diseases [3]. Consistent with previous studies, we noted that pleiotropic eQTLs are enriched in proximity to genes [5].

The differences between pleiotropic and non-pleiotropic eQTLs become evident at short distances. For example, the eQTL rs2522051 in the IL4 locus is associated with four trait categories, while nearby non-pleiotropic eQTLs (rs6894249, rs6894249, and rs80112473) at 31 bp, 656 bp, and 1795 bp distances are associated with fewer genes and tissues. The contrast is further illustrated by examples like rs159963 in the ENO1 locus and rs151174 in the SULT1A1 locus, where pleiotropic eQTLs exhibit more gene targets and tissue activity than their non-pleiotropic counterparts. Conversely, some pleiotropic eQTLs, such as rs823118 in the RAB29 locus, rs12656497 in the NPR3 locus, rs10051765 in the FRFR14 locus, region chr11:1,865,076–1,877,434 in the TNNT3 locus, rs2071382 in the FES locus, rs8067378 in the ORMDL3 locus, and rs601338 in the FUT2 locus. target fewer genes and tissues than non-pleiotropic counterparts, suggesting additional factors influencing eQTL pleiotropy remain to be identified.

Our study also shows that pleiotropic eQTLs are enriched in enhancer regions and transcription factor binding sites. This supports previous research indicating that pleiotropic enhancers, active across multiple tissues, comprise a small portion of the genome yet are enriched in transcription factor binding and gene regulatory connections [36].

Recent CRISPRi screens highlight that enhancer-target gene interactions are enriched within topological associated domains and CTCF ChIA-PET loops [22], [37], which could serve to get genes from GWAS variants within specific contexts [38]. In our findings, pleiotropic eQTLs and their target genes also exhibited enrichment in CTCF loops.

Our analysis reveals that pleiotropic eQTLs generally show reduced GWAS effect sizes (beta), greater significance in both eQTL and GWAS associations, and higher allele frequencies. Furthermore, pleiotropic eQTLs enriched in enhancers and transcription factor binding sites regulate a broader set of genes within CTCF loops.

## CRediT authorship contribution statement

**Pascale Paul:** Writing – review & editing, Software, Conceptualization. **Aitor González:** Writing – review & editing, Writing – original draft, Visualization, Validation, Software, Resources, Methodology, Formal analysis, Data curation, Conceptualization.

## Declaration of Generative AI and AI-assisted technologies in the writing process

During the preparation of this work the authors used https://chatgpt.com/ in order to improve language and readability. After using this tool/service, the authors reviewed and edited the content as needed and take full responsibility for the content of the publication.

## Declaration of Competing Interest

The authors declare that they have no known competing financial interests or personal relationships that could have appeared to influence the work reported in this paper.

## Data Availability

An interactive table for searching and browsing GWAS/eQTL colocalizations with PP.H4.abf≥ 0.75 is accessible through the website application: http://gwas2eqtl.tagc.univ-amu.fr/gwas2eqtl. The raw table of colocalizations with PP.H4.abf≥ 0.75 and the full colocalization data can be downloaded from https://doi.org/10.5281/zenodo.14064652.

## References

[1] Buniello A., MacArthur J.A.L., Cerezo M., Harris L.W., Hayhurst J., Malangone C. (2019). The NHGRI-EBI GWAS Catalog of published genome-wide association studies, targeted arrays and summary statistics 2019. Nucleic Acids Res.

[2] Andrews G., Fan K., Pratt H.E., Phalke N., Consortium Z., Karlsson E.K. (2023). Mammalian evolution of human cis-regulatory elements and transcription factor binding sites. Science.

[3] Shikov A.E., Skitchenko R.K., Predeus A.V., Barbitoff Y.A. (2020). Phenome-wide functional dissection of pleiotropic effects highlights key molecular pathways for human complex traits. Sci Rep.

[4] Jordan D.M., Verbanck M., Do R. (2019). HOPS: a quantitative score reveals pervasive horizontal pleiotropy in human genetic variation is driven by extreme polygenicity of human traits and diseases. Genome Biol.

[5] Watanabe K., Stringer S., Frei O., Umićević Mirkov M., de Leeuw C., Polderman T.J.C. (2019). A global overview of pleiotropy and genetic architecture in complex traits. Nat Genet.

[6] Chesmore K., Bartlett J., Williams S.M. (2018). The ubiquity of pleiotropy in human disease. Hum Genet.

[7] Barrio-Hernandez I., Schwartzentruber J., Shrivastava A., Del-Toro N., Gonzalez A., Zhang Q. (2023). Network expansion of genetic associations defines a pleiotropy map of human cell biology. Nat Genet.

[8] Mu Z., Wei W., Fair B., Miao J., Zhu P., Li Y.I. (2021). The impact of cell type and context-dependent regulatory variants on human immune traits. Genome Biol.

[9] Giambartolomei C., Vukcevic D., Schadt E.E., Franke L., Hingorani A.D., Wallace C. (2014). Bayesian test for colocalisation between pairs of genetic association studies using summary statistics. PLoS Genet.

[10] Pan S., Kang H., Liu X., Li S., Yang P., Wu M. (2024). COLOCdb: a comprehensive resource for multi-model colocalization of complex traits. Nucleic Acids Res.

[11] GTEx Consortium. The GTEx Consortium atlas of genetic regulatory effects across human tissues. Science 2020;369:1318–1330. https://doi.org/10.1126/science.aaz1776. 10.1126/science.aaz1776 PMC7737656 32913098

[12] Ribeiro D.M., Rubinacci S., Ramisch A., Hofmeister R.J., Dermitzakis E.T., Delaneau O. (2021). The molecular basis, genetic control and pleiotropic effects of local gene co-expression. Nat Commun.

[13] Kerimov N., Hayhurst J.D., Peikova K., Manning J.R., Walter P., Kolberg L. (2021). A compendium of uniformly processed human gene expression and splicing quantitative trait loci. Nat Genet.

[14] Lyon M.S., Andrews S.J., Elsworth B., Gaunt T.R., Hemani G., Marcora E. (2021). The variant call format provides efficient and robust storage of GWAS summary statistics. Genome Biol.

[15] Hammal F., de Langen P., Bergon A., Lopez F., Ballester B. (2022). ReMap 2022: a database of human, mouse, drosophila and arabidopsis regulatory regions from an integrative analysis of DNA-binding sequencing experiments. Nucleic Acids Res.

[16] Nassar L.R., Barber G.P., Benet-Pagès A., Casper J., Clawson H., Diekhans M. (2023). The UCSC genome browser database: 2023 update. Nucleic Acids Res.

[17] The ENCODE Project ConsortiumAbascal F., Acosta R., Addleman N.J., Adrian J., Afzal V. (2020). Expanded encyclopaedias of DNA elements in the human and mouse genomes. Nature.

[18] Mölder F., Jablonski K.P., Letcher B., Hall M.B., Tomkins-Tinch C.H., Sochat V. (2021). Sustainable data analysis with snakemake. F1000Res.

[19] Zhao H., Sun Z., Wang J., Huang H., Kocher J.-P., Wang L. (2014). CrossMap: a versatile tool for coordinate conversion between genome assemblies. Bioinformatics.

[20] 1000 Genomes Project Consortium, Auton A., Brooks L.D., Durbin R.M., Garrison E.P., Kang H.M. (2015). A global reference for human genetic variation. Nature.

[21] McLaren W., Gil L., Hunt S.E., Riat H.S., Ritchie G.R.S., Thormann A. (2016). The ensembl variant effect predictor. Genome Biol.

[22] Luo R., Yan J., Oh J.W., Xi W., Shigaki D., Wong W. (2023). Dynamic network-guided CRISPRi screen identifies CTCF-loop-constrained nonlinear enhancer gene regulatory activity during cell state transitions. Nat Genet.

[23] Chun S., Casparino A., Patsopoulos N.A., Croteau-Chonka D.C., Raby B.A., De Jager P.L. (2017). Limited statistical evidence for shared genetic effects of eQTLs and autoimmune-disease-associated loci in three major immune-cell types. Nat Genet.

[24] Connally N.J., Nazeen S., Lee D., Shi H., Stamatoyannopoulos J., Chun S. (2022). The missing link between genetic association and regulatory function. Elife.

[25] Jostins L., Ripke S., Weersma R.K., Duerr R.H., McGovern D.P., Hui K.Y. (2012). Host-microbe interactions have shaped the genetic architecture of inflammatory bowel disease. Nature.

[26] de Lange K.M., Moutsianas L., Lee J.C., Lamb C.A., Luo Y., Kennedy N.A. (2017). Genome-wide association study implicates immune activation of multiple integrin genes in inflammatory bowel disease. Nat Genet.

[27] Liu J.Z., van Sommeren S., Huang H., Ng S.C., Alberts R., Takahashi A. (2015). Association analyses identify 38 susceptibility loci for inflammatory bowel disease and highlight shared genetic risk across populations. Nat Genet.

[28] Reay W.R., Cairns M.J. (2021). Advancing the use of genome-wide association studies for drug repurposing. Nat Rev Genet.

[29] Schmiedel B.J., Singh D., Madrigal A., Valdovino-Gonzalez A.G., White B.M., Zapardiel-Gonzalo J. (2018). Impact of genetic polymorphisms on human immune cell gene expression. Cell.

[30] Fairfax B.P., Makino S., Radhakrishnan J., Plant K., Leslie S., Dilthey A. (2012). Genetics of gene expression in primary immune cells identifies cell type-specific master regulators and roles of HLA alleles. Nat Genet.

[31] Momozawa Y., Dmitrieva J., Théâtre E., Deffontaine V., Rahmouni S., Charloteaux B. (2018). IBD risk loci are enriched in multigenic regulatory modules encompassing putative causative genes. Nat Commun.

[32] Mouri K., Guo M.H., de Boer C.G., Lissner M.M., Harten I.A., Newby G.A. (2022). Prioritization of autoimmune disease-associated genetic variants that perturb regulatory element activity in T cells. Nat Genet.

[33] Schuch J.B., Polina E.R., Rovaris D.L., Kappel D.B., Mota N.R., Cupertino R.B. (2016). Pleiotropic effects of Chr15q25 nicotinic gene cluster and the relationship between smoking, cognition and ADHD. J Psychiatr Res.

[34] Liu H., Sun Y., Zhang X., Li S., Hu D., Xiao L. (2020). Integrated analysis of summary statistics to identify pleiotropic genes and pathways for the comorbidity of schizophrenia and cardiometabolic disease. Front Psychiatry.

[35] Arguinano A.-A.A., Ndiaye N.C., Masson C., Visvikis-Siest S. (2018). Pleiotropy of ABO gene: correlation of rs644234 with E-selectin and lipid levels. Clin Chem Lab Med.

[36] Singh D., Yi S.V. (2021). Enhancer pleiotropy, gene expression, and the architecture of human enhancer–gene interactions. Mol Biol Evol.

[37] Yao D., Tycko J., Oh J.W., Bounds L.R., Gosai S.J., Lataniotis L. (2024). Multicenter integrated analysis of noncoding CRISPRi screens. Nat Methods.

[38] Gschwind A.R., Mualim K.S., Karbalayghareh A., Sheth M.U., Dey K.K., Jagoda E., et al. An encyclopedia of enhancer-gene regulatory interactions in the human genome 2023. 10.1101/2023.11.09.563812. PMC13471189 42457959

