# Supplementary material for: Pleiotropic expression quantitative trait loci are enriched in enhancers and transcription factor binding sites and impact more genes
Source: Comput Struct Biotechnol J. 2024 Nov 17;23:4260–70. doi: 10.1016/j.csbj.2024.11.019 (PMC11635986; doi:10.1016/j.csbj.2024.11.019)
Supplement: Supplementary file 1 — Supplementary material [file mmc1.pdf]

Supplementary Information

Supplementary Figure 1a

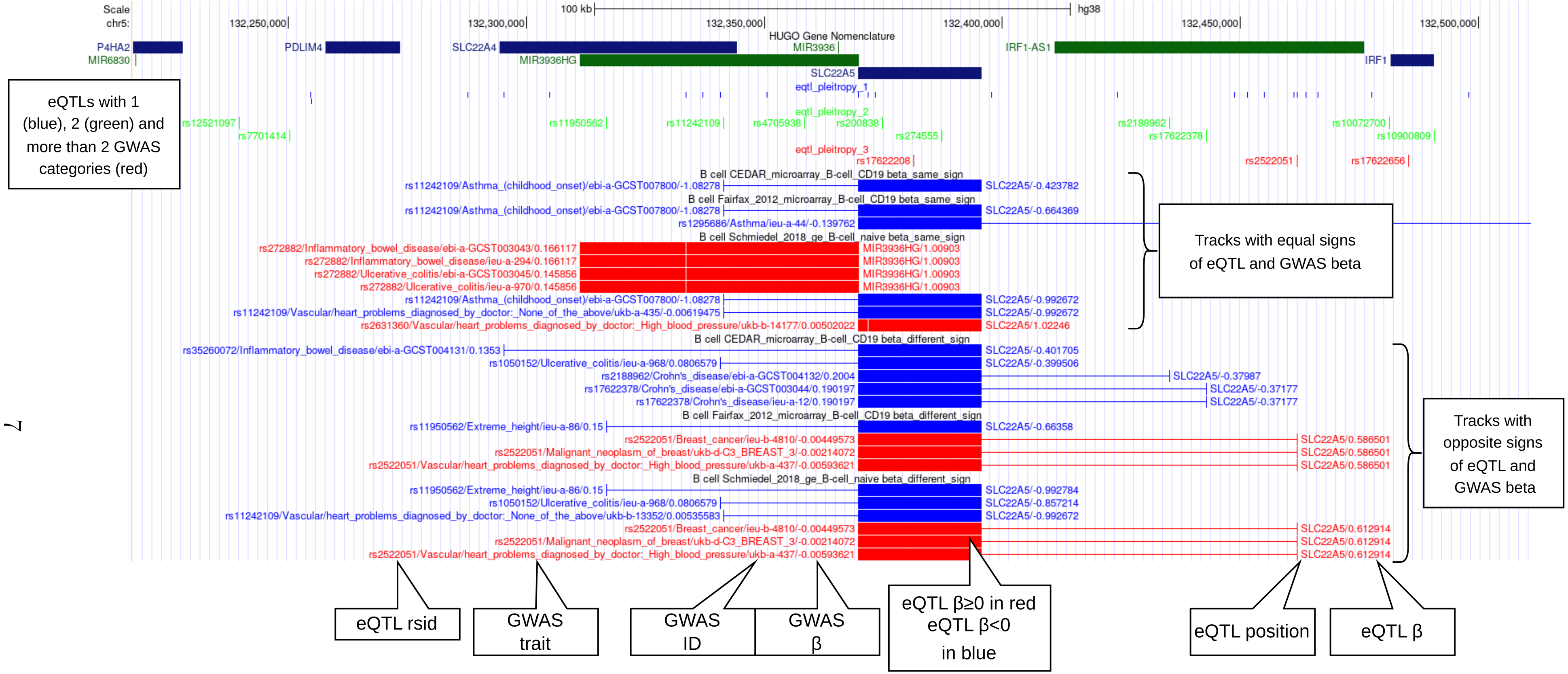

Supplementary Figure 1b

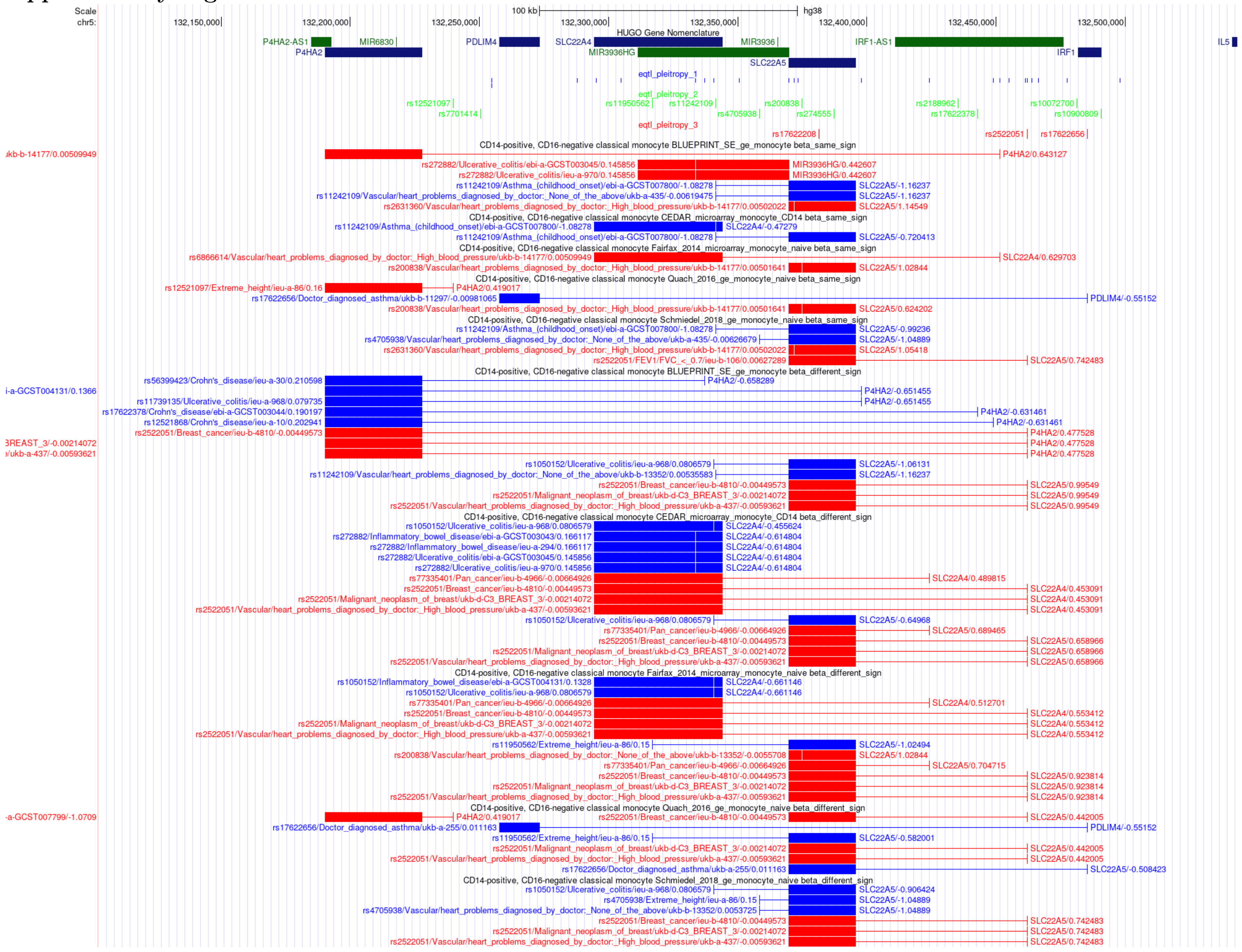

Supplementary Figure 1c

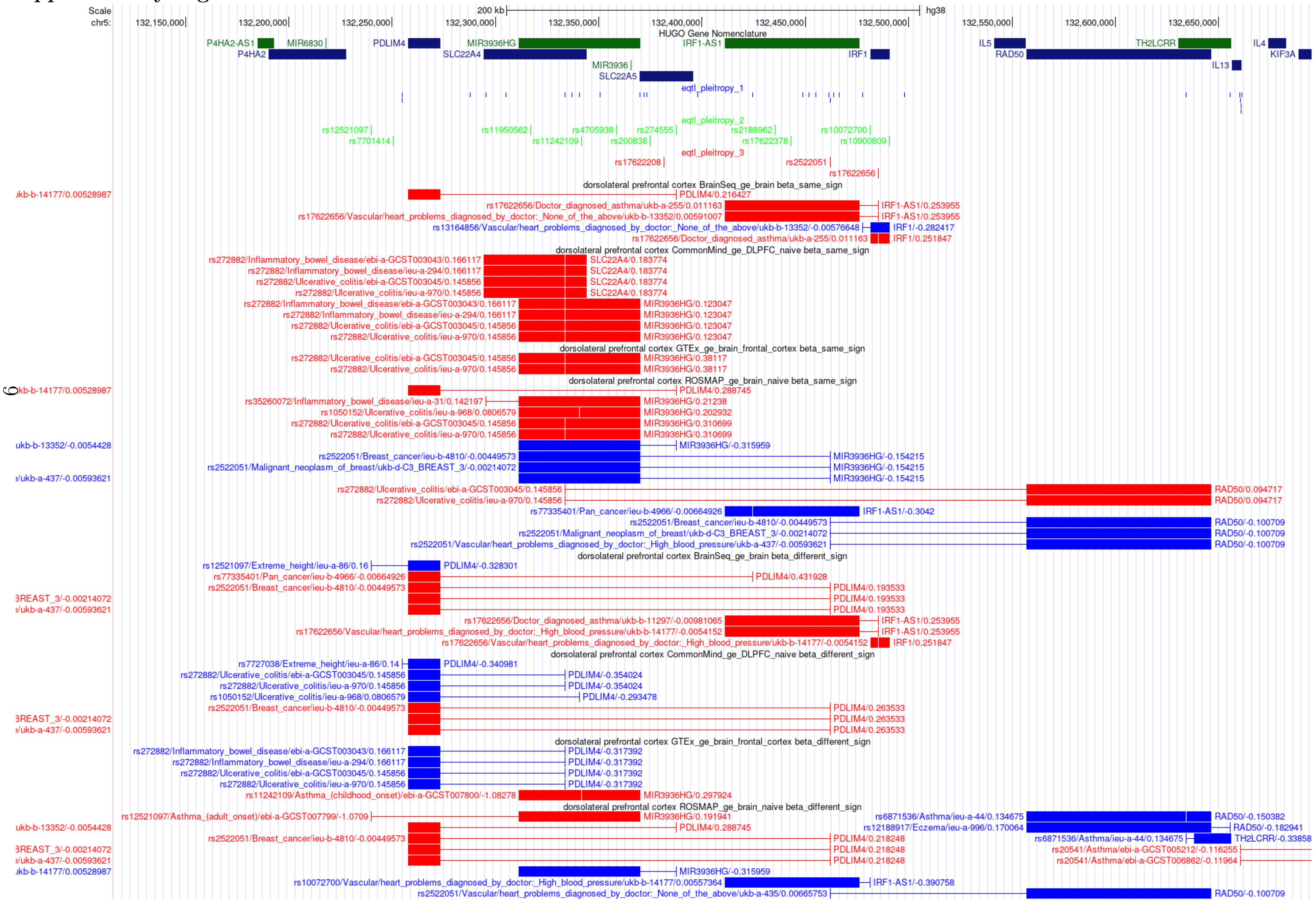

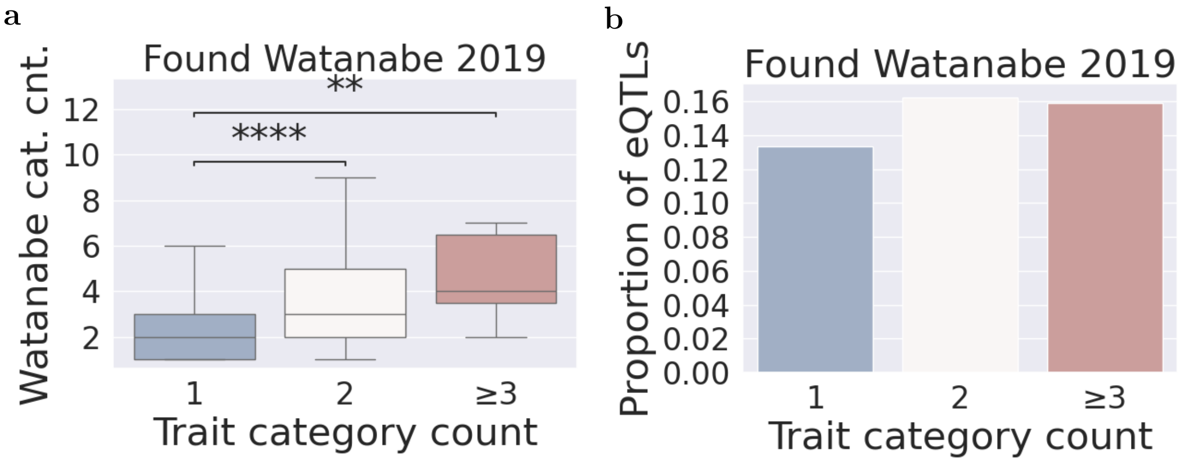

Supplementary Figure 2

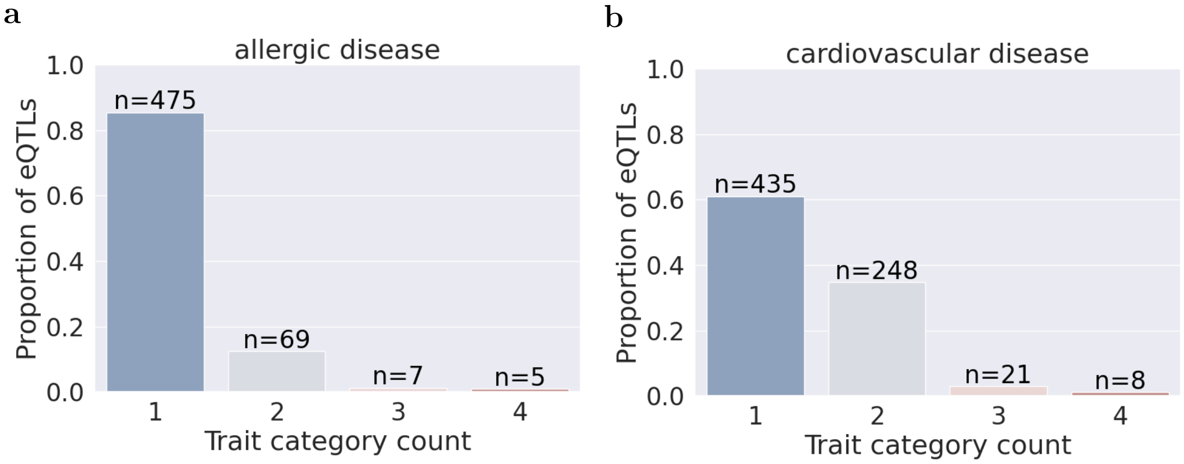

Supplementary Figure 3

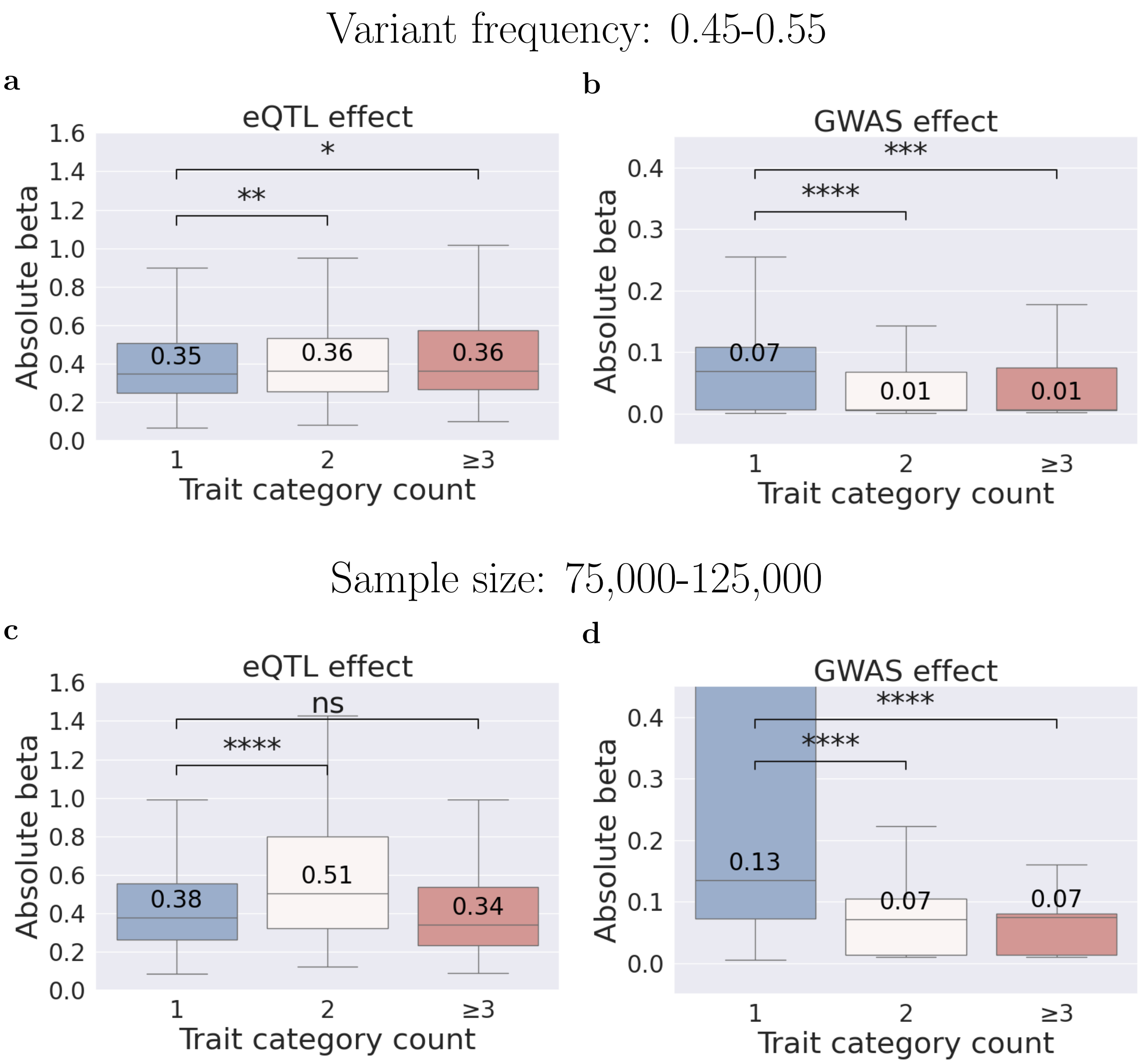

Supplementary Figure 4

Variant frequency: 0-1

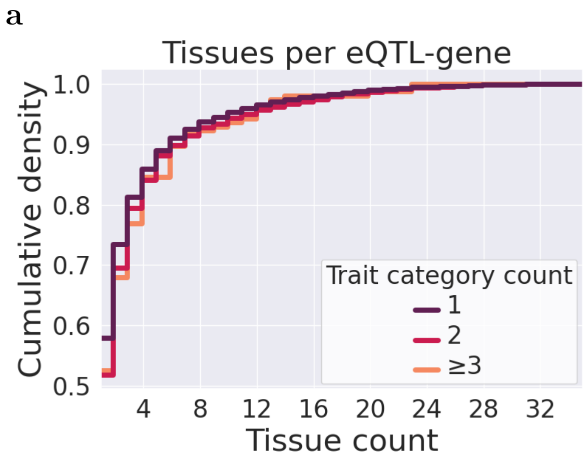

Variant frequency: 0.45-0.55

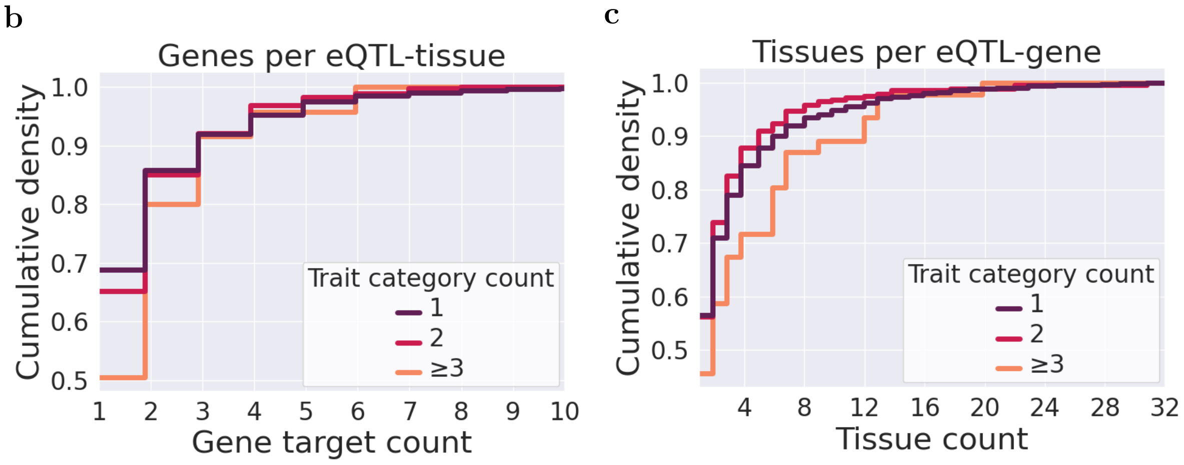

Supplementary Figure 5

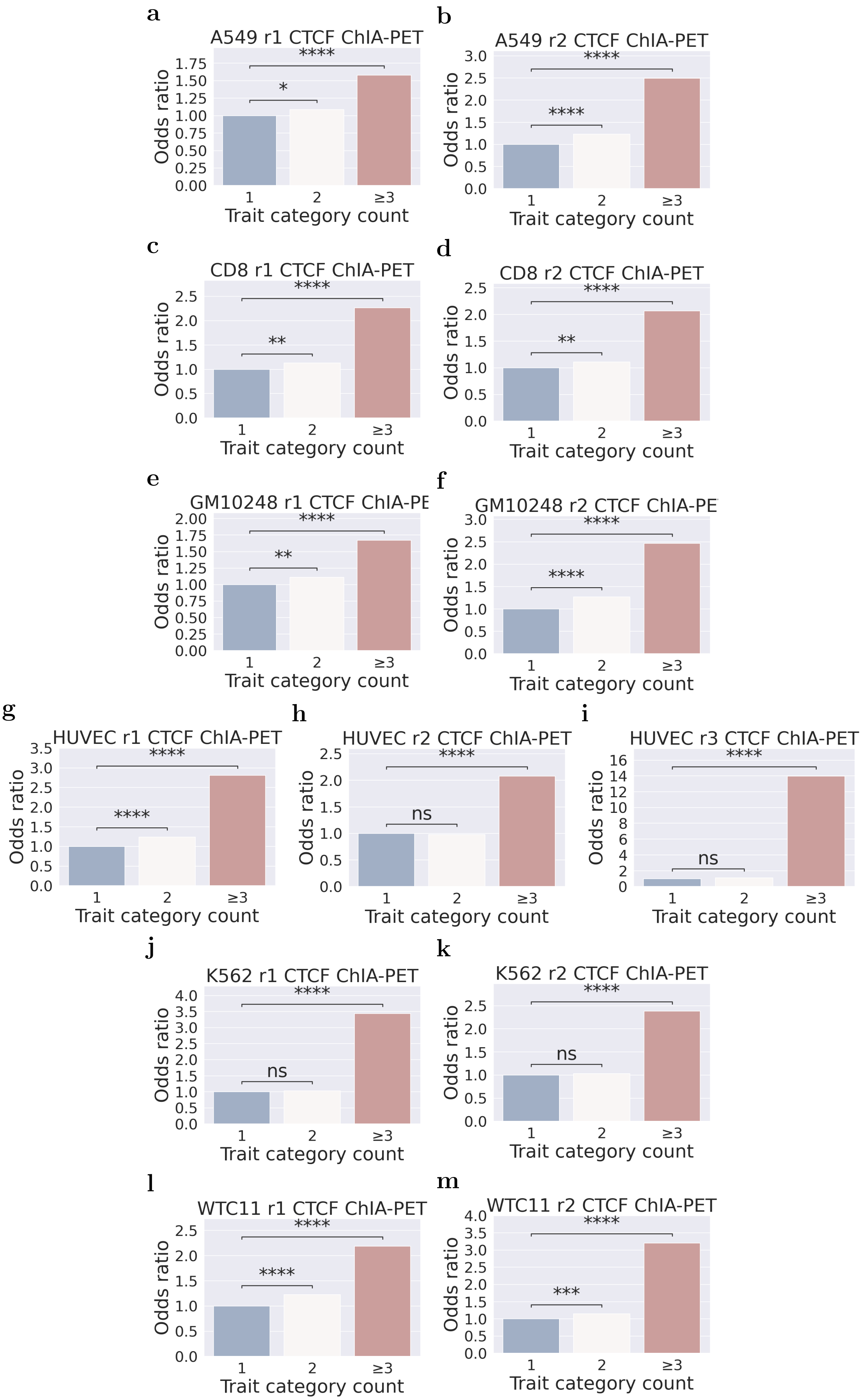

Supplementary Figure 6
