# Supplementary material for: Pleiotropic expression quantitative trait loci are enriched in enhancers and transcription factor binding sites and impact more genes
Source: Comput Struct Biotechnol J. 2024 Nov 17;23:4260–70. doi: 10.1016/j.csbj.2024.11.019 (PMC11635986; doi:10.1016/j.csbj.2024.11.019)
Supplement: Supplementary file 7 — Supplementary material [file mmc7.docx]

# Supplementary figure and table legends

**Supplementary Figure 1: UCSC tracks screenshot for eQTLs colocalizing with GWAS variants in the specified regions.**

screenshot for eQTLs colocalizing with GWAS variants in the specified region (chr5:132,137,990-132,728,110, hg38) for B-cells (a), classical monocytes (b), and cortex cells (c):

In panel (a), denoting B-cells, the image illustrates different components. The red color signifies positive eQTL beta coefficients, while blue represents negative eQTL beta coefficients. Two sections are provided for each biological sample: "beta equal" corresponds to eQTLs with the same effect sign as the GWAS, and "beta unequal" corresponds to eQTLs colocalizing with a GWAS but with a different effect sign. This distinction enables the inference of the beta coefficient of the GWAS variant based on the color of the eQTL beta.

For example, in B-cells (a), a decrease in the expression of the SLC22A5 gene is correlated with a decrease in Asthma predisposition and an increase in extreme height and inflammatory bowel diseases.

**Supplementary Figure 2: Comparison with the analysis by Watanabe et al 2019.**

a) Comparative analysis of trait categories counts in our study and that conducted by Watanabe et al. [5].

b) The percentage of our eQTLs in the study by Watanabe et al. is depicted for various pleiotropy levels [5]. The boxplots represent quartiles of the dataset, with whiskers extending to show the remainder of the distribution except for outliers.

In figure (a), we conducted the Mann-Whitney U test. Significance levels are denoted as follows: ns (not significant) for ≥0.05, * for <0.05, ** for <0.01, *** for <0.001, and **** for <0.0001.

**Supplementary Figure 3: Pleiotropic eQTL distribution in allergic and blood-related and cardiovascular diseases.**

These bar plots depict the proportions of eQTLs with different levels of pleiotropy that colocalize with allergic (a) and cardiovascular diseases (b). The values above the bar plots show the count of eQTLs.

**Supplementary Figure 4: eQTL and GWAS effects for given variant frequencies and sample sizes.**

Average of the absolute effect of variants on gene expression (a, c) and traits (b, d) for variants with frequencies in the interval between 0.45 and 0.55 (a, b) and samples size of the GWAS studies in the interval between 75,000 and 125,000 (c, d).

**Supplementary Figure 5: Distribution of tissue and gene count.**

(a, c) Cumulative proportion of tissue count per eQTL-gene pair with increasing counts of tissues for unconstrained variant frequency (a) and variant frequencies between 0.45 and 0.55. (b) Cumulative proportion of gene count per eQTL-tissue pair with increasing counts of genes for variant frequencies between 0.45 and 0.55.

**Supplementary Figure 6: Enrichment of pleiotropic eQTLs within CTCF ChIA-PET loops.**

These bar plots depict the odds ratio of pleiotropic eQTLs within CTCF ChIA-PET loops in isogenic replicates r1 (a, c, e, g, j, l), r2 (b, d, f, h, k, m) and r3 (i) of cell lines A549 (a, b), CD8 T-cells (c, d), GM10248 (e, f), HUVEC (g-i), K562 (j, k) and WTC11 (l-m).

**Supplementary Table 1: List of eQTL studies and annotations.**

This tables shows additional annotation regarding tissue category terms to complement the information derived from eQTL studies in the EBI eQTL Catalogue.

**Supplementary Table 2: List of GWAS studies and annotations.**

The 417 GWAS studies in our analysis have been annotated with both a GWAS trait ontology and GWAS trait category information. This additional annotation provides a more comprehensive understanding of the traits associated with the genetic variations studied in the GWAS datasets.

**Supplementary Table 3: Proportion of leading SNPs in GWAS studies explained by colocalized eQTLs.**

This table provides the percentage of leading SNPs in GWAS that colocalize with eQTLs.

**Supplementary Table 4: List of 5,345 GWAS variants that colocalize with eQTLs at PP.H4.abf≥0.75 and SNP.H4.PP≥ 0.5 with annotations.**

This table offers the list of 5,345 GWAS variants that colocalize with eQTLs at PP.H4.abf≥0.75 and SNP.H4.PP≥ 0.5 with annotations, including details such as the most cited eQTL gene and its number of Pubmed citations (Columns H, I), trait category and trait counts (Columns J, K), a list of trait categories, trait ontology, and eQTL gene identifier and symbols (Columns L, M), eQTL gene identifier and symbols, and the number of eQTL genes (Columns N-P), the biological sample category list and their numbers (Columns Q, R), the most cited gene identifier (Column S), and the number of domains (equivalent to trait categories) in [5] (Column T).

**Supplementary Table 5: List of genomic regions containing colocalized eQTLs and GWAS variants.**

This table contains the full list of regions, including information on the number and list of trait category counts (Columns E, F), the symbol and identifier of eQTL genes (Columns G, I), and the category of the tissue (Column H). The number of trait categories is determined by the count of different trait categories in the region[18].
